# Editorial for Special Issue “Advances in Public Health and Healthcare Management for Chronic Care”

**DOI:** 10.3390/medicina61101771

**Published:** 2025-09-30

**Authors:** Fabio Petrelli, Giovanni Cangelosi

**Affiliations:** School of Pharmacy, Experimental Medicine and “Stefania Scuri” Public Health Department, University of Camerino, Via Madonna delle Carceri 9, 62032 Camerino, Italy

The management of chronic diseases represents one of the foremost challenges for contemporary healthcare systems, demanding increasingly innovative approaches and multidimensional strategies to improve outcomes for patients, families, and their communities [1]. This Special Issue, entitled “Advances in Public Health and Healthcare Management for Chronic Care,” brings together original studies and reviews that offer fresh perspectives on epidemiology, clinical management, and organizational models, thereby reinforcing the value of an integrated and multidisciplinary vision of chronic conditions.

Among the original contributions, Al-Dayan [2] analyzed the distribution of hemoglobin-related disorders, underscoring the strategic role of systematic screening and genetic counseling in identifying at-risk groups. Gitto et al. [3] investigated the application of artificial neural networks to distinguish between idiopathic normal pressure hydrocephalus and Alzheimer’s dementia, highlighting the potential of advanced technologies to enhance diagnostic accuracy. Park et al. [4] examined adiposity trends among peri- and postmenopausal women, while, in a complementary study, Park et al. [5] analyzed sales patterns of osteoporosis medications, offering valuable insights for health policy and resource allocation.

Borze Ursu et al. [6] assessed the effects of home-based relaxation compared with standard care in patients with fibromyalgia, reporting significant improvements in physiological parameters and suggesting complementary strategies for symptom management. Alruwaili [7] explored knowledge levels and therapeutic adherence among hypertensive patients, identifying key behavioral and educational determinants. Matsugaki et al. [8] investigated the relationship between a cancer diagnosis and the intention to leave the workforce in older individuals, shedding light on the broader social and occupational implications of chronic disease.

The second section of this Special Issue features reviews, beginning with the scoping review by Cangelosi et al. [9], which examined barriers and facilitators to the implementation of artificial intelligence in diabetes management from the perspective of healthcare professionals, emphasizing both the opportunities and challenges of digital innovation. Azzellino et al. [10] analyzed organizational models and the role of family and community nurses in the management of chronic conditions, underscoring the importance of integrated approaches.

The two systematic reviews by Karibayeva et al. [11,12] addressed vitamin D deficiency in Kazakhstan, focusing, respectively, on pediatric and adult populations. Considering both groups provides complementary evidence on the prevalence of a widespread condition, highlighting the importance of preventive strategies across the life course.

Taken together, the contributions included in this Special Issue illustrate the inherent complexity of chronic care, spanning epidemiological, clinical, social, and organizational dimensions. They also underscore the potential of innovative methodologies and interdisciplinary approaches to enhance patient outcomes and overall health system performance. Ultimately, this collection reinforces the vision of chronic care as a domain where clinical, technological, and organizational innovation converge in the pursuit of the most effective and personalized strategies for individuals, communities, and populations, with a strong emphasis on prevention [13].
